# The CHANSE score: a novel clinical tool incorporating the heel drop test for the diagnosis of pediatric appendicitis

**DOI:** 10.3389/fped.2025.1724592

**Published:** 2025-12-15

**Authors:** Min Kyo Chun, Jun Sung Park, Dahyun Kim, Jeeho Han, Jeong-Yong Lee, Jong Seung Lee, Seung Jun Choi

**Affiliations:** 1 University of Ulsan College of Medicine, Songpa-gu, Republic of Korea; 2 Department of Pediatrics, Asan Medical Center, Songpa-gu, Republic of Korea; 3 Department of Emergency Medicine, Asan Medical Center, Songpa-gu, Republic of Korea

**Keywords:** appendicitis, clinical scoring system, heel drop test, pediatric, emergency department (ED)

## Abstract

**Background:**

Acute appendicitis is one of the most common abdominal surgical emergencies in children presenting to the emergency department. Although clinical guidelines emphasize combining symptom characteristics, physical examination findings, and inflammatory markers for accurate diagnosis, pediatric presentations are often atypical and overlap with other conditions. Classic physical signs such as the obturator, psoas, and Rovsing's signs have limited predictive value. The heel drop test has been reported to outperform rebound tenderness in detecting intraperitoneal inflammation, as it is more objective and less susceptible to misinterpretation. However, evidence regarding its diagnostic utility in appendicitis is limited, and its clinical application in pediatric patients has not been specifically examined.

**Objective:**

To evaluate the clinical utility of the heel drop test for diagnosing appendicitis in children and to develop a novel clinical scoring system that incorporates this test.

**Methods:**

We conducted a prospective observational study at a tertiary pediatric emergency center between August 2021 and August 2023. Children with suspected appendicitis underwent standardized clinical and laboratory evaluation, including the heel drop test. Variables significantly associated with appendicitis were identified and used to create a new scoring system (CHANSE: CRP elevation, Heel drop test positivity, Anorexia, Nausea or vomiting, Shift to the left, Elevated WBC). Diagnostic performance was assessed and compared with the Pediatric Appendicitis Score (PAS) using ROC analysis.

**Results:**

Among 142 enrolled children, 84 were diagnosed with appendicitis. A positive heel drop test was significantly more common in the appendicitis group and showed diagnostic performance comparable to cough/percussion/hopping tenderness. The CHANSE score demonstrated superior diagnostic accuracy compared with PAS (AUC 0.794 vs. 0.763, *p* < 0.001). A CHANSE score ≥3 showed predictive characteristics similar to a PAS cutoff ≥7. Higher CHANSE scores were also associated with complicated appendicitis.

**Conclusion:**

The heel drop test is a useful and objective physical examination method for diagnosing pediatric appendicitis. The CHANSE score, which incorporates this test, is a simple and reliable tool with diagnostic performance comparable to or better than PAS. It may also assist in identifying complicated appendicitis and support timely clinical decision-making.

## Introduction

Acute appendicitis is one of the most common abdominal surgical emergencies among children presenting to the emergency department (1). Recent clinical guidelines emphasize that a combination of symptom characteristics, physical examination findings such as tenderness, and laboratory evidence of inflammation can enable accurate diagnosis in the majority of suspected appendicitis cases (2). However, the clinical presentation of appendicitis in children is often atypical, as symptoms may overlap with or be accompanied by those of other conditions (3). Moreover, when patient cooperation is limited, it may be challenging to perform a reliable physical examination (4). Classic physical signs such as the obturator sign, psoas sign, and Rovsing's sign have limited predictive value for appendicitis (5).

The heel drop test has been reported to be superior to rebound tenderness for detecting intraperitoneal inflammation, as it is more objective and less prone to misinterpretation (6). Nevertheless, few studies have evaluated the diagnostic utility of the heel drop test in appendicitis (7, 8),. While recent reports have suggested the possible diagnostic role of the hopping test (9), to date, no study has specifically examined the clinical application of the heel drop test in pediatric populations. An alternative diagnostic strategy involves the use of clinical scoring systems, the most widely used being the Pediatric Appendicitis Score (PAS) (10, 11). This score is calculated based on symptoms, physical examination findings, and laboratory results, with a total score ranging from 0 to 10; a score of 7 or higher is considered to have high predictive value for appendicitis (12).

The primary aim of this study is to evaluate the diagnostic utility of the heel drop test, previously validated in adults, for diagnosing acute appendicitis in children. To this end, we compare the performance of the heel drop test with well-established clinical parameters to assess its diagnostic accuracy. Additionally, we propose a new clinical scoring system incorporating the heel drop test as a variable and evaluate its reliability by comparing it with the existing PAS.

## Materials and methods

### Study design and population

This prospective observational study was conducted at the Pediatric Emergency Center of Asan Medical Center Children's Hospital in Seoul, Korea, between August 2021 and August 2023. Pediatric patients under 18 years of age who presented to the emergency department with abdominal pain were screened for eligibility. Among them, patients were included if they exhibited right lower abdominal pain accompanied by clinical signs or symptoms suggestive of acute appendicitis, or if they were transferred from other hospitals with suspected appendicitis. The study excluded children younger than 5 years of age, those unable to comply with examination instructions, and those with a previous appendectomy.

### Definition of variables

Demographic, clinical, and laboratory parameters were recorded prospectively, including the results of the heel drop test, physical findings, and laboratory values such as white blood cell (WBC) count, neutrophil percentage, and C-reactive protein (CRP) level. The presence of anorexia, nausea or vomiting, diarrhea, migration of pain was also documented. A comprehensive set of physical examination findings was systematically collected. These examinations were performed in a sequence as follows.

The heel drop test was performed with the patient standing on their toes while facing the emergency physician conducting the test. The patient was instructed to drop their heels abruptly to the floor using their full body weight. A positive test was defined as the occurrence of abdominal pain, facial expression changes suggestive of discomfort, groaning, or protective movements such as clutching the abdomen or bending the waist during the maneuver (7, 8). Following the heel drop test, the patient was asked to cough and hop to assess for elicited tenderness, which was documented accordingly.

Afterward, the patient was placed in a supine position for a physical examination. Right lower quadrant (RLQ) tenderness, RLQ rebound tenderness, the psoas sign, obturator sign, and Rovsing's sign were assessed and recorded In cases with clinical suspicion of appendicitis, contrast-enhanced abdominal computed tomography (CT) was performed, and the final diagnosis was confirmed by a radiologist. Cases showing findings such as perforated appendicitis, periappendicular abscess, or peritonitis were classified as complicated appendicitis.

### Statistical analysis and score development

Statistical analysis was performed using SPSS software, version 21.0 (SPSS Inc., Chicago, IL). Categorical variables were presented as frequency and percentages, while continuous variables were expressed as mean ± standard deviation. Differences between the appendicitis and non-appendicitis groups were evaluated using univariate analysis. A *p*-value of less than 0.05 was considered statistically significant.

Variables demonstrating statistically significant associations in univariate analysis were subsequently incorporated into the construction of the CHANSE score. The optimal cutoff value for predicting appendicitis was determined using the Youden Index, which was derived from receiver operating characteristic (ROC) curve analysis and represents the point that maximizes the sum of sensitivity and specificity.

The diagnostic performance of the CHANSE score was compared with that of the Pediatric Appendicitis Score (PAS) through ROC curve analysis and calculation of standard diagnostic metrics. The difference between the areas under the curve (AUCs) of the CHANSE and PAS scores was assessed using a nonparametric *Z*-test for correlated ROC curves, based on the estimated standard error of the AUC difference. Sensitivity, specificity, positive predictive value (PPV), negative predictive value (NPV), positive likelihood ratio (PLR), and negative likelihood ratio (NLR) were calculated for both scoring systems. As all analyses were performed within the same derivation cohort without external validation. This study was approved by the Institutional Review Board of Asan Medical Center.

## Results

**Figure 1:**
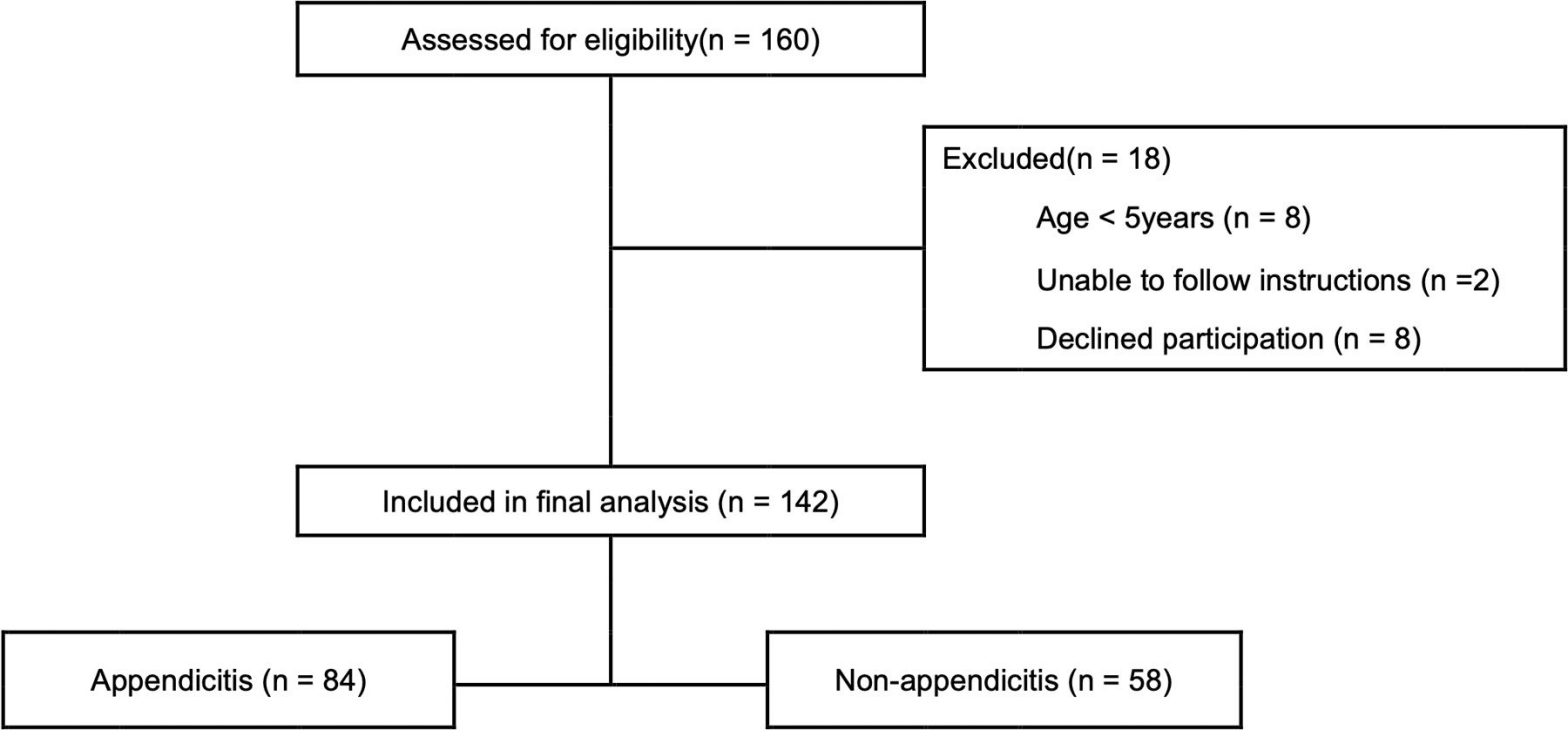
Flow diagram of patient enrollment, exclusions,and final diagnostic classification.

A total of 160 patients were screened during the study period. Of these, 18 were excluded – 8 were under 5 years of age, 2 were unable to follow instructions, and 8 declined participation—resulting in 142 pediatric patients included in the final analysis. Among the 84 patients diagnosed with appendicitis (Figure 1), 10 were treated with antibiotics alone, whereas the remaining patients underwent surgical appendectomy. The proportion of male patients was higher in the appendicitis group, although there was no significant difference in age between the groups. There were no significant differences in the presence of migratory pain, fever, or diarrhea. However, anorexia and nausea or vomiting were significantly more common in the appendicitis group.

Among laboratory findings, elevated white blood cell (WBC) counts, neutrophilia, and increased C-reactive protein (CRP) levels were significantly associated with appendicitis. Regarding physical examination findings, RLQ tenderness did not differ significantly between groups, while tenderness elicited by coughing, percussion, or hopping was more frequently observed in the appendicitis group. A positive heel drop test was also significantly more common among patients with appendicitis (Table 1).

**Table 1:** Demographic and clinical data for the study population.

Features (n, %)	Appendicitis (*n* = 84, 59.2%)	No appendicitis (*n* = 58, 40.8%)	p	Odd ratio	95% CI
Age (years)	11.3 ± 2.9	12.0 ± 3.1	0.160	0.883	0.454–1.718
Male gender (%)	52 (61.9)	24 (41.4)	0.018	2.302	1.162–4.559
Body temperature	37.3 ± 0.7	37.1 ± 0.6	0.216	1.013	0.962–1.067
Temperature ≥ 38.0℃	35 (41.7)	20 (34.5)	0.109	1.357	0.678–2.716
Duration of pain (hr)	27.6 ± 22.1	27.3 ± 20.2	0.202	1.010	0.986–1.025
Anorexia	49 (58.3)	20 (34.5)	0.006	2.660	1.329–5.323
Nausea or vomiting (%)	47 (56.0)	8 (13.8)	<0.001	7.939	3.354–18.794
Diarrhea (%)	19 (22.6)	16 (27.6)	0.577	0.767	0.355–1.657
Migration(%)	22 (26.2)	15 (25.9)	0.932	1.017	0.474–2.181
WBC (x10^3^/µL)	13.7 ± 5.3	9.6 ± 3.9	<0.001	1.001	1.001–1.002
Leukocytosis ≥ 10,000/µL	61 (72.6)	22 (37.9)	<0.001	4.340	2.123–8.872
Neutrophil (%)	77.9 ± 14.6	63.6 ± 15.4	<0.001	1.047	1.013–1.083
PMN ≥ 75%	59 (70.2)	16 (27.6)	<0.001	6.195	2.950–13.008
CRP (mg/dL)	4.3 ± 9.1	1.3 ± 2.9	0.019	1.087	1.006–1.175
CRP ≥ 5 mg/dL	22 (26.2)	5 (8.6)	0.009	3.761	1.332–10.620
Tenderness in RLQ	72 (85.7)	43 (74.1)	0.086	2.093	0.896–4.887
Cough/percussion/hopping tenderness	68 (81.0)	39 (67.2)	0.042	2.071	1.056–4.484
Psoas	27 (32.1)	16 (27.6)	0.529	1.243	0.596–2.595
Rovsing	25 (30.0)	7 (12.1)	0.009	3.087	1.233–7.732
Obturator	31 (36.9)	18 (31.0)	0.377	1.300	0.638–2.647
Heel drop	66 (78.6)	35 (60.3)	0.024	2.410	1.149–5.053

WBC, white blood cell; PMN, polymorphonucleocytes; CRP, C-reactive protein, RLQ, right lower quadrant.

Variables that showed statistically significant differences across symptoms, laboratory findings, and physical examinations were incorporated into a new scoring system, named the CHANSE score, consisting of the following components: CRP elevation, Heel drop test positivity, Anorexia, Nausea or vomiting, Shift to the left, and Elevated WBC count. Each variable was assigned 1 point, yielding a total possible score of 0–6 (Table 2).

**Table 2:** Development of the CHANSE score.

Variable	Point
CRP ≥ 1 mg/dL	1
Heel drop test	1
Anorexia	1
Nausea or vomiting	1
Shift to left	1
Elevated WBC	1

**Figure 2:**
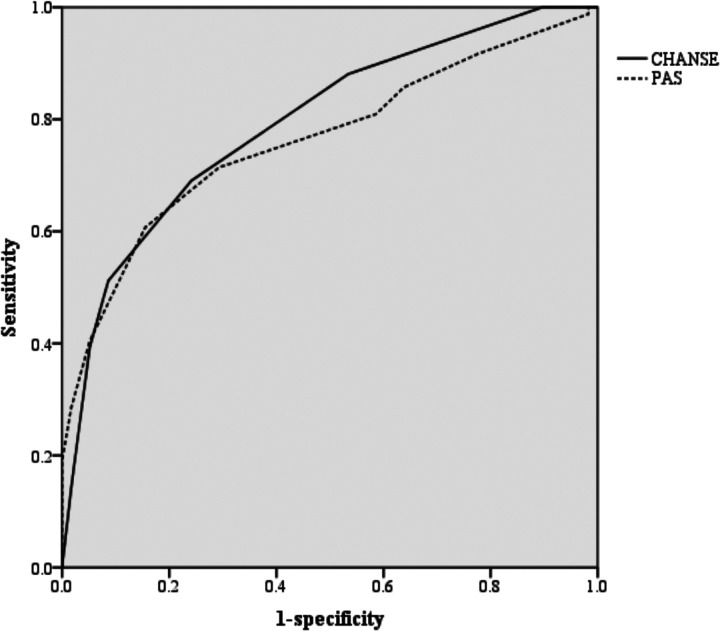
Receiver operating characteristic (ROC) curve analysis for the CHANSE score and the PAS. CHANSE CRP, Heel drop test, Anorexia, Nausea or vomiting, Shift to left, Elevated WBC PAS Pediatric Appendicitis Score.

The diagnostic performance of the CHANSE score was compared to the Pediatric Appendicitis Score (PAS) using receiver operating characteristic (ROC) curve analysis. The CHANSE score demonstrated a higher area under the curve (AUC) than the PAS: AUC 0.794 (95% CI, 0.720–0.867) vs. AUC 0.763 (95% CI, 0.685–0.840), respectively; *p* < 0.001 (Figure 2).

Among physical examination findings, the heel drop test showed diagnostic performance comparable to cough/percussion/hopping tenderness and demonstrated higher sensitivity than laboratory parameters such as leukocytosis, neutrophilia, and elevated CRP levels (Table 3).

**Table 3:** Performance of the parameters included in the PAS, and CHANSE score.

Parameters	Sensitivity	Specificity	PPV	NPV	PLR	NLR	p
Migration	0.27 (0.17–0.37)	0.74 (0.61–0.85)	0.59 (0.45–0.72)	0.41 (0.37–0.46)	1.02 (0.58–1.80)	0.99 (0.81–1.21)	0.932
Anorexia	0.58 (0.47–0.69)	0.66 (0.52–0.78)	0.71 (0.62–0.78)	0.52 (0.44–0.60)	1.69 (1.14–2.52)	0.64 (0.46–0.87)	0.006
Nausea or vomiting	0.56 (0.45–0.67)	0.86 (0.75–0.94)	0.85 (0.75–0.92)	0.57 (0.51–0.64)	4.06 (2.07–7.93)	0.51 (0.39–0.66)	<0.001
Tenderness in RLQ	0.86 (0.76–0.92)	0.26 (0.15–0.39)	0.63 (0.58–0.67)	0.56 (0.39–0.71)	1.16 (0.97–1.38)	0.55 (0.28–1.09)	0.086
Cough/percussion/hopping tenderness	0.81 (0.71–0.89)	0.33 (0.21–0.46)	0.64 (0.59–0.68)	0.54 (0.40–0.68)	1.22 (1.02–1.48)	0.56 (0.33–0.98)	0.042
Fever (≥ 38.0℃)	0.14 (0.8–0.24)	0.91 (0.81–0.97)	0.71 (0.47–0.87)	0.42 (0.40–0.45)	1.66 (0.62–4.45)	0.94 (0.83–1.06)	0.109
Leukocytosis(≥ 10,000/µL)	0.73 (0.62–0.82)	0.62 (0.48–0.74)	0.73 (0.66–0.80)	0.61 (0.51–0.70)	1.91 (1.34–2.73)	0.44 (0.30–0.66)	<0.001
Neutrophilia(≥ 75%)	0.70 (0.59–0.80)	0.72 (0.59–0.83)	0.79 (0.70–0.85)	0.63 (0.54–0.71)	2.55 (1.64–3.95)	0.41 (0.29–0.59)	<0.001
CRP(≥ 1 mg/dL)	0.62 (0.51–0.72)	0.62 (0.48–0.74)	0.70 (0.62–0.77)	0.53 (0.45–0.61)	1.63 (1.13–2.36)	0.61 (0.44–0.86)	0.006
Heel drop	0.79 (0.68–0.87)	0.40 (0.27–0.53)	0.65 (0.60–0.70)	0.56 (0.43–0.68)	1.30 (1.03–1.65)	0.54 (0.32–0.91)	0.024

RLQ, right lower quadrant; CRP, C-reactive protein PPV, positive predictive value, NPV, negative predictive value; PLR, positive likelihood ratio; NLR, negative likelihood ratio; PAS, Pediatric Appendicitis Score, CHANSE, Cough/Hopping/Anorexia/Neutrophilia/Heel-drop/Shift-to-left Evaluation.

Based on ROC analysis, a CHANSE score cutoff of 3 demonstrated predictive characteristics comparable to a PAS cutoff of 7 (Table 4).

**Table 4:** Evaluation of the performance of PAS and the CHANSE score indicative of appendicitis.

Score threshold	Sensitivity	Specificity	PPV	NPV	PLR	NLR
CHANSE ≥ 2	0.88 (0.79–0.94)	0.47 (0.33–0.60)	0.70 (0.65–0.75)	0.73 (0.59–0.84)	1.65 (1.28–2.12)	0.26 (0.13–0.49)
CHANSE ≥ 3	0.69 (0.58–0.79)	0.76 (0.63–0.86)	0.81 (0.72–0.87)	0.63 (0.54–0.71)	2.86 (1.77–4.61)	0.41 (0.29–0.58)
CHANSE ≥ 4	0.51 (0.40–0.62)	0.91 (0.81–0.97)	0.90 (0.78–0.95)	0.56 (0.51–0.62)	5.94 (2.50–14.08)	0.53 (0.42–0.67)
PAS ≥ 6	0.81 (0.71–0.89)	0.41 (0.29–0.55)	0.67 (0.61–0.72)	0.60 (0.47–0.72)	1.38 (1.09–1.76)	0.46 (0.27–0.79)
PAS ≥ 7	0.71 (0.61–0.81)	0.71 (0.57–0.82)	0.78 (0.70–0.84)	0.63 (0.54–0.71)	2.44 (1.60–3.72)	0.40 (0.28–0.59)
PAS ≥ 8	0.61 (0.49–0.71)	0.84 (0.73–0.93)	0.85 (0.75–0.91)	0.60 (0.53–0.66)	3.91 (2.10–7.31)	0.47 (0.35–0.62)

PPV, positive predictive value; NPV, negative predictive value; PLR, positive likelihood ratio; NLR, negative likelihood ratio.

**Figure 3:**
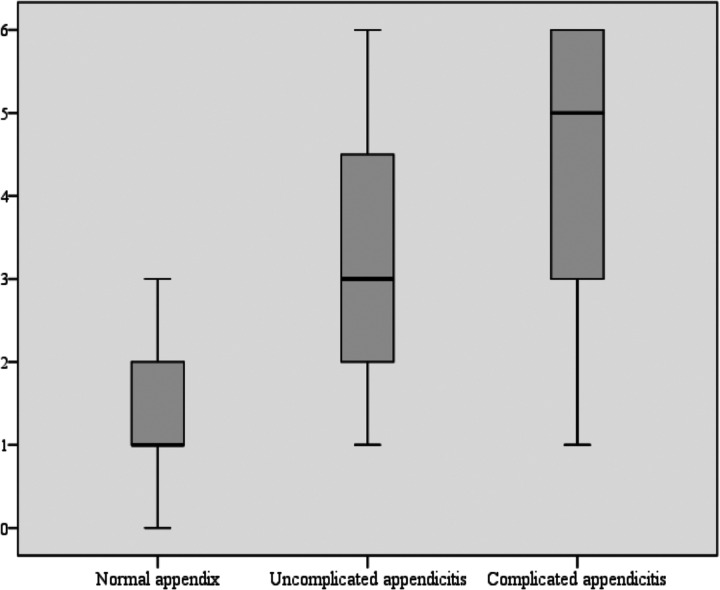
Box plot displaying the distribution of the CHANSE score in patients with a normal appendix, uncomplicated appendicitis, and complicated appendicitis. CHANSE CRP, Heel drop test, Anorexia, Nausea or vomiting, Shift to left, Elevated WBC.

Among the 84 patients diagnosed with appendicitis, 52 had uncomplicated appendicitis and 32 had complicated appendicitis. The mean CHANSE score was significantly higher in the complicated appendicitis group compared to the uncomplicated group (Figure 3).

## Discussion

This study demonstrated that the heel drop test is a useful physical examination tool for diagnosing appendicitis in pediatric patients, similar to its proven utility in adults (8). Moreover, clinical features such as anorexia, nausea or vomiting, leukocytosis, neutrophilia, and elevated CRP levels were significantly more common in children with appendicitis. When these variables, including the heel drop test, were incorporated into a scoring system, the diagnostic performance was found to be comparable to or superior to that of the widely used Pediatric Appendicitis Score (PAS).

Traditionally, physical examination findings suggestive of appendicitis include right lower quadrant (RLQ) tenderness, rebound tenderness, the psoas sign, obturator sign, and Rovsing's sign (5). Although some studies have reported these signs to be useful in pediatric patients (13), others have questioned their diagnostic value due to low sensitivity (14). In our study, the psoas and obturator signs did not differ significantly between groups, and while Rovsing's sign was more frequently observed in the appendicitis group, it had a low positivity rate (30%), indicating limited sensitivity. Although RLQ tenderness was more common among children with appendicitis (85.7% vs. 74.1%), the difference was not statistically significant (*p* = 0.086). This may be due to the frequent absence of these signs in younger children (15) and the presence of RLQ tenderness in other conditions such as mesenteric lymphadenitis, reducing its specificity (16).

In contrast, the heel drop test was significantly more likely to be positive in patients with appendicitis and demonstrated diagnostic performance comparable to cough/percussion/hopping tenderness, a parameter included in PAS. These findings support the use of the heel drop test as a reliable physical examination method in pediatric appendicitis, just as in adults.

Interestingly, migratory pain—another component of PAS—did not differ significantly between the appendicitis and non-appendicitis groups in our study. This aligns with previous reports indicating that up to one-third of children with appendicitis do not exhibit migratory pain (17). The concept of pain migration may be difficult for young children to understand, recall accurately, or articulate clearly, which may explain its limited diagnostic value in pediatric populations.

Regarding laboratory findings, leukocytosis and neutrophilia were significantly more common in the appendicitis group, consistent with prior research. Elevated CRP levels were also more frequent, justifying their inclusion in the new scoring system. The Appendicitis Inflammatory Response (AIR) score includes leukocytosis, neutrophilia, and CRP, and has been shown to be useful in diagnosing appendicitis (18). Some studies have reported that the AIR score may outperform the Alvarado score in diagnostic accuracy (19, 20). Our study selected only those PAS variables that showed significant differences in our dataset, combined with CRP as an inflammatory marker, to create a simpler, faster, and more effective scoring system—CHANSE—that matched or exceeded the diagnostic performance of PAS.

This study has several strengths. First, the CHANSE score incorporates the heel drop test, a more objective and less interpretation-dependent examination method compared to traditional physical signs (6). Classic physical examinations often require children to lie down, which can be difficult in anxious, crying, or uncooperative children. The heel drop test, however, is simple to perform, can be done in a playful manner at a distance, and may help reduce patient anxiety. Its assessment criteria—such as vocal expressions of pain, facial grimacing, and protective postures—are more objective and less reliant on clinician experience or child cooperation. Additionally, including CRP alongside leukocytosis and neutrophilia provides a more robust and quantifiable marker of inflammation.

Second, the CHANSE score uses fewer variables than PAS, each assigned a score of 1, making it easier to memorize and calculate in clinical settings. A score of ≥3 out of 6 correlates with the high-risk category of PAS (≥7), providing a simple and practical threshold for clinical decision-making.

Furthermore, compared with the Pediatric Appendicitis Score (PAS) and the Appendicitis Inflammatory Response (AIR) score, the CHANSE score streamlines decision-making by using fewer equally weighted variables. This simplicity allows rapid bedside assessment and facilitates more consistent, timely decisions in busy pediatric emergency settings.

Third, in addition to diagnosing acute appendicitis, the CHANSE score may aid in identifying cases of complicated appendicitis. A higher CHANSE score was associated with complicated disease, suggesting its potential utility in guiding treatment decisions, such as the need for urgent surgical intervention. In our cohort, patients with uncomplicated and complicated appendicitis showed progressively increasing CHANSE scores (3.0 ± 1.5 vs. 4.6 ± 1.5), indicating a clear trend between score elevation and disease severity. However, because the number of patients with complicated appendicitis was limited (*n* = 32), the dataset was not sufficient to derive a statistically robust cutoff value for predicting complicated disease using ROC-based methods. Nevertheless, the observed distribution suggests that higher CHANSE scores may still serve as a practical framework for risk stratification, and future multicenter studies with larger sample sizes will be necessary to validate this potential application.

This study has several limitations. First, it included only children who were clinically suspected of having appendicitis at initial presentation. Given that pediatric appendicitis can present with non-specific signs and symptoms, it is possible that some cases were missed due to initial diagnostic uncertainty. However, no patients who were excluded from enrollment but presented with abdominal pain, nausea, or vomiting were later diagnosed with appendicitis on point-of-care ultrasound (POCUS) or CT imaging.

Second, the study included only children aged 6–17 years who were able to understand and follow instructions; younger children were excluded. Nonetheless, since the peak incidence of pediatric appendicitis occurs during the second decade of life (4, 21), the findings are still highly applicable in clinical practice.

Third, this study was conducted in a single tertiary center, and no external validation was performed. Therefore, further multicenter studies with larger cohorts are warranted to verify the generalizability and clinical robustness of the CHANSE score.

## Conclusion

In this study, we demonstrated the clinical utility of the heel drop test as a physical examination method for diagnosing appendicitis in pediatric patients. Furthermore, we developed a novel clinical scoring system incorporating the heel drop test and validated its diagnostic performance by comparison with the widely used Pediatric Appendicitis Score (PAS). Our findings suggest that this new score may serve as a practical and reliable tool for improving diagnostic accuracy in children with suspected appendicitis.

## Data Availability

The raw data supporting the conclusions of this article will be made available by the authors, without undue reservation.
